# Biocontrol Effects of *Paecilomyces variotii* against Fungal Plant Diseases

**DOI:** 10.3390/jof7060415

**Published:** 2021-05-26

**Authors:** Alejandro Moreno-Gavíra, Fernando Diánez, Brenda Sánchez-Montesinos, Mila Santos

**Affiliations:** Departamento de Agronomía, Escuela Superior de Ingeniería, Universidad de Almería, 04120 Almería, Spain; alejanmoga@gmail.com (A.M.-G.); fdianez@ual.es (F.D.); brensam@hotmail.com (B.S.-M.)

**Keywords:** *Paecilomyces*, biological control, endophyte, phytopathogens, aerial and soil diseases

## Abstract

The genus *Paecilomyces* is known for its potential application in the control of pests and diseases; however, its use in agriculture is limited to few species. Research interest in new formulations based on microorganisms for the control of pathogens is growing exponentially; therefore, it is necessary to study new isolates, which may help control diseases effectively, and to examine their compatibility with established agricultural control methods. We analysed in vitro and in vivo the antagonistic capacity of *Paecilomyces variotii* against seven phytopathogens with a high incidence in different crops, and we examined its compatibility with 24 commercial fungicides. *P. variotii* was applied in the following pathosystems: *B. cinereal—*melon, *Sclerotinia sclerotiorum—*pepper, *R. solani*—tomato, *F. solani—*zucchini, *P. aphanidermatum—*melon, *M. melonis*—melon, and *P. xanthii—*zucchini. The results showed strong control effects on *M. melonis* and *P. xanthii*, reducing the disease severity index by 78% and 76%, respectively. The reduction in disease severity in the other pathosystems ranged from 29% to 44%. However, application of metabolites alone did not cause any significant effect on mycelial growth of phytopathogens, apart from *F. solani*, in which up to 12% inhibition was observed in vitro when the extract was applied at a concentration of 15% in the medium. *P. variotii* was compatible with most of the tested fungicides, and of the 24 fungicides tested at the maximum authorised dose, 6 acted as fungicides, 4 as fungistatics, and the remaining showed inhibition rates ranging from 18.2% to 95.8%. These results indicate that *P. variotii* is a potential biological control agent to be used against several aerial and soil diseases, thus it should be integrated into modern pest management strategies.

## 1. Introduction

Various pests and diseases cause significant annual losses in global agriculture by reducing yield and crop quality [1,2] and such losses are estimated at around 20–40%, which represents a significant economic impact valued at hundreds of millions of dollars per year [3,4]. Common practice for controlling such pathogens is the indiscriminate application of agrochemicals; however, biological control by endophytic microorganisms is a more environmentally friendly alternative [5,6,7].

The endophytic fungus *Paecilomyces variotii* is an ascomycete that is morphologically characterised by the development of verticillate conidiophores with irregular branches in which cylindrical phialides are inserted with a pronounced narrowing at the neck where yellow or pale brown conidia are located [8,9]. This fungus produces chlamydospores with walls that are smooth or slightly rough, depending on the strain. *P. variotii* is thermophilic, ubiquitous, and highly versatile, and its spores can withstand temperatures above 80 °C for 15 min [10]; furthermore, it grows rapidly, can develop under oxygen-limited conditions, and produces a wide range of metabolites, therefore this species stands out among decomposing fungi [11]. It can adapt to a large variety of habitats (in air, edaphic, and aquatic environments) and can be found in plant debris and compost, inside plants as an endophyte, and in animals as an opportunistic parasite, thereby contaminating human food and animal feed; furthermore, it occurs in municipal solid waste, in sediments and marine plants, and even within homes and operating rooms [12,13,14,15,16,17,18].

The potential of *P. variotii* and of its bioactive metabolites for controlling fungal diseases has been extensively studied [19]. This species can reduce mycelial development of numerous phytopathogens under in vitro culture conditions. In particular, *P. variotii* can inhibit the fungi *Aspergillus niger* [20], *Botrytis cinerea* [21], *Fusarium graminearum* [22], *F. moniliforme* [23], *F. oxysporum* [24,25,26], *F. solani* [27], *Macrophomina phaseolina* [27,28], *Magnaporthe oryzae* [29], *Pyricularia oryzae* [30], *Pythium aphanidermatum* [31,32], *P. spinosum* [33], *Saccharomyces cerevisiae* [30], *Sclerotium rolfsii* [27,33], and *Verticillium dahilae* [25], and the bacteria *Bacillus cereus* [34], *B. subtilis* [20], *Escherichia coli*, *Micrococcus luteus* [22,35], *Pseudomonas aeruginosa* [30,34], *Staphylococcus aureus* [22,23,24,25,26,27,28,29,30,31,32,33,34,35], *Streptococcus iniae* [35], *Vibrio parahaemolyticus* [22,34], and *Xanthomonas campestris* [24], among others.

Similarly, the in vivo biocontrol effects on certain pathogens by *P. variotii* has been described in field experiments in which it significantly reduced the incidence and severity of diseases in different crops. Such treatments resulted in significant improvements of germination proportion, length and weight of roots and aerial plant parts, and yield [26]. Taken together, *P. variotii* exhibits a positive effect when inoculated together with *Alternaria alternata* [36], *Fusarium* spp. [37], *F. oxysporum* [24,26], *M. phaseolina* [28,37], *P. syringae* [38], *Rhizoctonia solani* [37], *P. aphanidermatum* [32], *X. campestris* [24], and *X. oryzae* [38].

The use of endophytic microorganisms has the advantage of being safe for humans; however, they may occasionally elicit various adverse effects such as phytotoxicity or environmental dependence for adequate biological activity [23], which is why the use of bioactive extracts and metabolites of these endophytes, including *P. variotii*, is of considerable interest [39]. A multitude of compounds synthesised by this fungus and able to act as microbial control agents has been described, such as 6-decyl salicylic acid [40], phenopicolinic acid [25], eicosenoic acid [41], ascofuranone [42], betulin [34], sphingofungin E and F [43,44], lawsozaheer [45], paecilaminol [20], paecilocin A-D [40], paeciloxazine [44,46], peptaibols [47], chitinases [39], varioxepine A and B [22,48], viriditin A and B [49], viriditoxin, semi-viriditoxin, and semi-viriditoxic acid [16,23,50].

The *P. variotii* isolate CDG33 has recently been characterised as a plant growth promoter [51]. The aims of the current study were (1) to determine in vitro antagonistic effects of *P. variotii* CDG33 and extracts thereof on different agriculturally important phytopathogens; (2) to examine its in vivo capacity for controlling the development of diseases caused by such phytopathogens in different pathosystems under greenhouse conditions; and (3) to assess the compatibility of *P. variotii* with different fungicides commonly used for fungal disease control.

## 2. Materials and Methods

### 2.1. Fungal Isolates

*P. variotii* CDG33 was obtained from autochthonous plants grown at the Cabo de Gata Natural Park, Almería, Spain [51]. This isolate has been tested to determine its potential biocontrol effect against *Botrytis cinerea* Pers, *Sclerotinia sclerotiorum* (Lib.) de Bary, *Fusarium solani* (Mart.) Sacc, *Pythium aphanidermatum* (Edson) Fitzp, *Rhizoctonia solani* J.G. Kühn, *Mycosphaerella melonis* (Pass.) and *Podosphaera xanthii* (Castagne) U. Braun & Shishkoff.

Different crop plants infected with pathogens were collected in the province of Almería, Spain, during 2017–2019. Selected stem and leaf sections with active lesions were cut, and tissue sections from the boundaries between healthy and discoloured areas were placed in potato dextrose agar (PDA; Bioxon, Becton Dickinson, Mexico). Pathogenicity tests were carried out to confirm pathogenic capacity.

The fungal isolate was grown and maintened on PDA in the dark at 25 – 27 ± 2 °C for 5 or 10 days. Spore suspensions of *P. variotii* and phytopathogen isolates (except for *R. solani*, *S. sclerotiorum*, and *P. xanthii*) were prepared by flooding plates of 10-day-old cultures with sterile distilled water, scraping the surface using a sterile glass rod, and subsequent filtering of the suspensions, after which they were adjusted to a concentration of 1 × 10^6^ spores/mL using a Neubauer haemocytometer. In the case of *R. solani* and *S. sclerotiorum*, inoculation was performed using mycelium adjusted to a concentration of approximately 10^4^ colony forming units/mL (CFU·mL^−1^). To produce mycelium, two agar plugs were cut from a colony and were placed in a flask containing PDB. The flasks were placed on a shaker for four days at 25 °C, and the content was homogenised for subsequent inoculation. The inoculum of *P. xanthii* was directly collected from affected leaves.

### 2.2. Dual Culture Bioassays

*P. variotii* was screened for in vitro antagonism against six phytopathogens using the confrontation assay of Santos et al. [52]. Petri dishes (9 cm diameter) containing 17 mL PDA were prepared, and 0.5 cm plugs of mycelium of all fungi were cut from the growing edge of 7-days-old cultures with active growth of each isolate. The plugs were placed at the margin of Petri dishes with a distance of 8 cm between the antagonist and phytopathogen fungi. A plug of PDA medium was used as a control treatment while the pathogen plug was placed at the other side. All plates were sealed using parafilm and were incubated in the dark at 25 °C for different periods of time, depending on the growth rate of the respective pathogen (3–10 days).

Radial fungal colony growth was measured daily. Results were transformed into percentages of mycelium growth inhibition (PIRM). These tests were carried out using five replicates. The areas where antagonist and pathogen merged were also observed using a dissection microscope and a compound light microscope at 100-fold and 400-fold magnification.

### 2.3. Effects of Non-Volatile Metabolites of P. variotii on Fungal Pathogens

To determine the effects of non-volatile metabolites of *P. variotii*, 300 mL PDB medium was inoculated with two 0.5 cm plugs of mycelium and incubated without shaking at 25 °C in the dark for 7, 14, 21, 28, 58, 88, and 118 days. Subsequently, the medium was filtered through double layered muslin cloth to remove mycelium and was sterilized by microfiltration through sterilized membranes of 0.22 µm pore size (Merck Millipore, Burlington, MA, USA). Extracts were then incorporated and mixed with cooled PDA at 5, 10, and 15% (*v/v*) and were immediately poured into Petri dishes (50 mm). In the control treatments, distilled water was used instead of extract. Agar plugs (0.5 cm diameter) covered with actively growing mycelia of the different pathogens were individually inoculated on the media and were incubated in the dark at 25 °C for 10 days, apart from *P. aphanidermatum* which were incubated for 3 days, and *S. sclerotiorum* and *B. cinerea* which were incubated for 5 days under the same conditions. After incubation, mycelial growth of each fungus was measured as the average of two perpendicular diameters of the colony, and inhibitor effects were calculated in comparison to the controls. Five replicates were used for each incubation time and pathogen.

### 2.4. Antifungal Volatile Organic Compounds Bioassay

In vitro inhibition effects of volatile organic compounds produced by *P. variotii* against different phytopathogenic fungi was determined. For this, a plug (0.5 cm diameter) was cut from the actively growing edges of *P. variotii* plates and was placed at the centre of 5.5 cm Petri dishes containing PDA medium. After two days of incubation of the antagonist, the same procedure was repeated for each of the pathogens, and a Cartesian axis was drawn from the centre of each plate to facilitate measurements. Both open plates were placed on the base of a square 12 cm Petri dish on which a further inverted base was placed after which they were sealed using parafilm. The air volume was approximately 403.20 cm^3^, preventing direct contact between the two fungi. The control plates were prepared following the same procedure but without the antagonist plug. The plates were incubated at 25 ± 1 °C, and mycelial growth of the pathogen was measured in both directions, which was performed daily for fungi with rapid growth (*B. cinerea*, *S. sclerotiorum*, *P. aphanidermatum* and *R. solani*) and every two days for those growing more slowly (*M. melonis* and *F. solani*), until the edge of the plate was reached. Three replicates were examined each day.

### 2.5. Detached Leaf Assay

Before the in vivo assay, suppressive effects of *P. variotii* on *B. cinerea, S. sclerotiorum*, and *M. melonis* were assessed using a detached leaf assay as described by Novak et al. [53] and Patial et al. [54]. Leaves of cucumber (variety *Marketmore)*, pepper (var. red cherry) and tomato (var. Largo de Reus) seedlings were disinfected using 3% sodium hypochlorite for 30 s and were washed twice to remove residues. The leaves were immersed for 3 min in a solution containing *P. variotii* spores at a dose of 10^6^ spores·mL^−1^ or were immersed in an aqueous solution containing the fungicide Switch (cyprodinil 37.5% and fludioxonil 25% (WG) *w/w*; Syngenta, Basel, Switzerland) at 600 ppm, to compare efficacy. Whole leaves or fragments were then placed on wet filter paper in Petri dishes, and the centre of the leaf or leaf fragment was carefully punctured using a sterilised needle. A 0.5 cm plug containing the corresponding pathogen was placed at the puncture site. Petri dishes were then incubated at 25 °C, and number of leaves with symptoms were counted and photographed 72 h after inoculation. This experiment was repeated twice.

### 2.6. Compatibility of P. variotii with Fungicides

In vitro compatibility of *P. variotii* with different selected fungicides for mycelial growth inhibition was established using the poisoned food technique [55,56]. The minimum recommended dose (D2), the maximum recommended dose (D3), 0.5 × D2, and 1.5 × D3 (Table 1) of each fungicide were tested. Using a sterile cork borer, mycelial plugs (0.5 cm diameter) were cut from actively growing seven-day-old fungal cultures and were placed in the centre of a Petri dish containing PDA supplemented with various pesticides. Five replicates were used per treatment. Fungicidal or fungistatic effects of each fungicide was determined by transferring the initial plug of *P. variotii* which did not grow with fungicide, to a PDA plate without fungicide.

Toxicity, i.e., compatibility of *P. variotii* and the fungicide, was classified using the scale of the International Organisation for Biological Control (OILB) [57]. This classification groups the compatibility between microorganisms and fungicides, depending on the proportion of inhibition compared to a control (<30%: harmless; 30–75%: slightly toxic; 75–90%: moderately toxic; >90%: toxic).

### 2.7. Greenhouse Evaluation of P. variotii on Disease Severity of Seven Phytopathogens

Biocontrol effects of *P. variotii* on different pathosystems was determined: *B. cinereal—*melon (var. Piel de Sapo), *S. sclerotiorum—*pepper (var. Red Cherry), *R. solani—*tomato (var. Largo de Reus), *F. solani—*zucchini (*P. aphanidermatum—*melon, *M. melonis—*melon, and *P. xanthii—*zucchini (var. Milenio). Seeds were disinfected using 2% hypochlorite for 3 min and were washed thoroughly with tap water to eliminate residues. Subsequently, the seeds were sown in 500 mL pots containing a commercial peat mix at one seed per pot. A 5 mL spore suspension of each isolate of *P. variotii* (PAE) was added to each pot at 1 × 10^6^ propagules/plant; the control treatments received 5 mL water. To assess diseases of aerial parts, foliar spraying was carried out at the same dose to wet the whole plant with the *P. variotii* solution. Spraying was carried out three days before applying conidia/mycelium of the respective pathogen.

**Figure 1:**
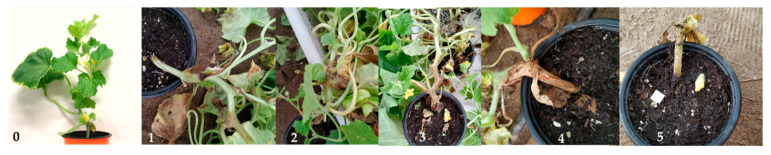
Example of the disease severity scale for *M. melonis.* **0** = healthy plant; **1** = initial symptoms; **2** = moderate symptoms (25%); **3** = affected plant (50%); **4** = severely affected plant (75%); **5** = dead plant.

To prepare *P. aphanidermatum* inocula the procedure described by Marin et al. [58] was followed. Inocula of the other phytopathogens were prepared by scraping and subsequent filtration as previously described, apart from *R. solani*, *S. sclerotiorum* and *P. xanthii*. Phytopathogens were inoculated when the plants showed a second true leaf, and using a sterile micropipette, inoculation was performed by uniformly applying the zoospores/conidia suspension (5 mL) at a concentration of 10^4^ CFU·mL^−1^ uniformly to the peat surface. In the case of *B. cinerea* and *M. melonis*, the pathogen was applied by spraying the plant five times at the same concentration (6 mL). Before this, the first true leaf had been cut to facilitate pathogen entry. Inoculation with *R. solani* was carried out by mixing mycelium into the substrate; inoculation with *S. sclerotiorum* was performed using the spray mycelium method as described by Chen and Wang [59]. The stem was wounded to facilitate pathogen entry. Symptom severity was recorded continuously, and 30–60 days after inoculation, a final disease severity index was estimated according to the following scale: 0 = healthy plant; 1 = initial symptoms; 2 = moderate symptoms (25%); 3 = affected plant (50%); 4 = severely affected plant (75%); and 5 = dead plant (Figure 1).

**Figure 2:**
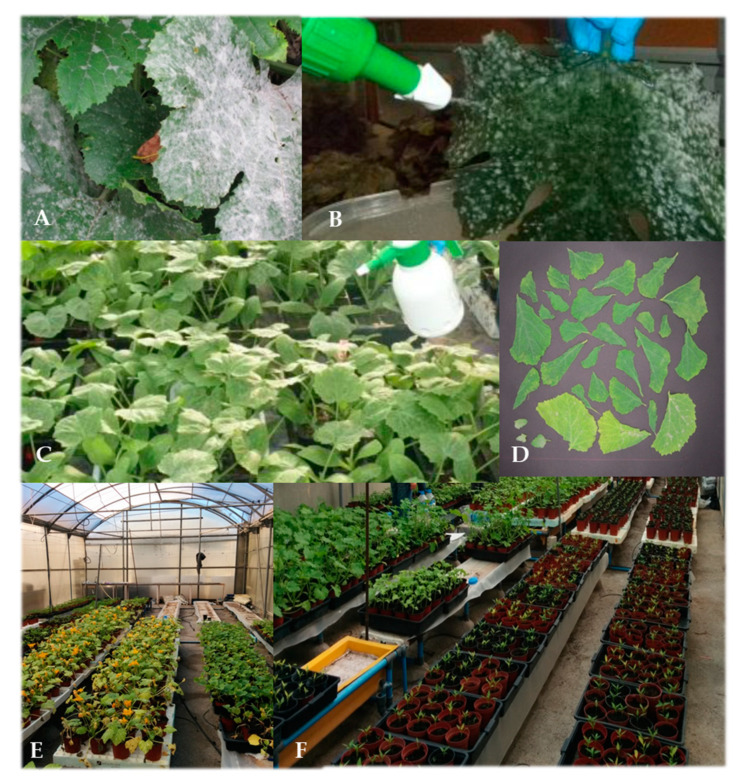
(**A**). Zucchini leaves with symptoms of powdery mildew caused by *P. xanthii*. (**B**). Application of sterile water for rinsing off spores. (**C**). Spraying of spores on healthy seedlings previously treated with *P. variotii* (PAE) or water (T0). (**D**). Arrangement of leaves to determine the affected leaf area. (**E**,**F**). Distribution of seedlings during the different assays.

To determine the control effect of *P. variotii* on powdery mildew, an inoculum of *P. xanthii* was prepared from field-collected zucchini leaves affected by cucurbit powdery mildew (Figure 2). Using a sprayer, sterile distilled water was sprayed under pressure to rinse the leaves of fungal conidia. The suspension was collected, and experimental plants were immediately inoculated at a concentration of 10^4^ CFU·mL^−1^. To determine the suppressive effect of *P. variotii* on the disease, the leaf area (Figure 2) affected by powdery mildew was determined using WinDIAS 3.1.lnk software (Dynamax, Fresno, CA, USA) to calculate the proportion of affected leaf area with respect to total leaf area. Additionally, the numbers of affected leaves and petioles per plant were counted. 

All pathogenicity tests were performed under greenhouse conditions and in different seasons to provide optimal environmental conditions for each pathogen. Experimental units consisted of four repetitions with 24 plants per pathosystem. Two experiments were conducted using a completely randomized block design.

### 2.8. Statistical Analyses

Data were analysed using analysis of variance with Statgraphics Centurion version XVI software. A preliminary test was performed to assess the normal distribution of the data. Mean separation was carried out using Fisher’s least significant difference test. Data were tested using a one-way analysis of variance or Student’s *t*-test; statistical significance is reported at *p* < 0.05.

## 3. Results

### 3.1. In Vitro Antagonism of P. variotii

**Figure 3:**
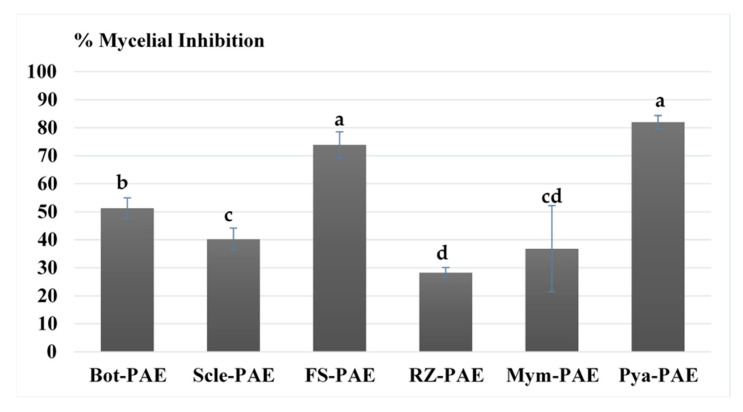
Antagonistic effects of Paecilomyces variotii (PAE) on Botrytis cinerea (Bot), Sclerotinia sclerotiorum (Scle), Fusarium solani (FS), Pythium aphanidermatum (Pya), Rhizoctonia solani (Rz), and Mycosphaerella melonis (Mym) in dual culture on PDA medium. Percent mycelial inhibition was calculated as PIRM = ((R1 − R2)/R1) × 100, where PIRM is the percentage of inhibition of radial mycelia growth of the pathogen. R1 is radial growth of the pathogen in control plates, and R2 is radial growth of the pathogen in dual cultures. Means with the same letter are not significantly different (least significant difference test) according to an analysis of variance (*p* > 0.05).

**Figure 4:**
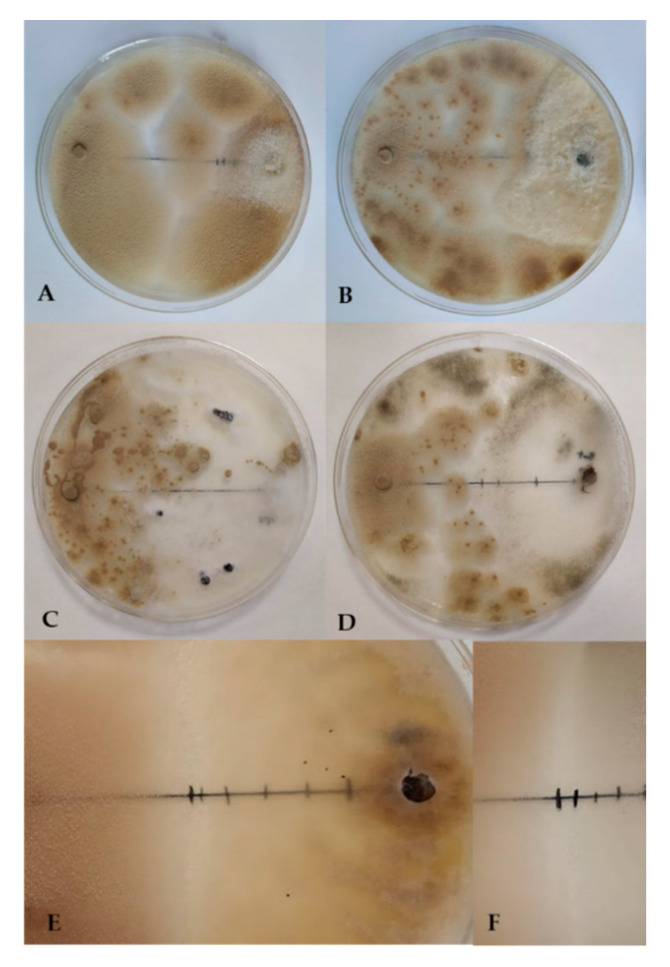
In vitro antagonistic effects of *P. variotii* on *F. solani* (**A**), *M. melonis* (**B**), *S. sclerotiorum* (**C**), and *B. cinerea* (**D**) in 9 cm Petri dishes. Inhibition zone between *P. variotii* and the phytopathogens *M. melonis* (**E**) and *F. solani* (**F**) (Images 75% increased).

The results of the dual culture assay, expressed as inhibition percentages, are shown in Figure 3. Colony growth of *P. variotii* occurred rapidly (48–72 h) with isolated colonies covering the plate, demonstrating the inhibitory effect of the antagonist (Figure 4). The highest percentages of inhibition were detected in *P. aphanidermatum* and *F. solani*. Moreover, *P. variotii* initially showed inhibition of approximately 2 mm (Figure 4), which subsequently disappeared under direct contact between the two colonies. This inhibition zone indicates the presence of fungistatic metabolites secreted by *P. variotii*. Parasitism was not observed, even though hyphal destruction and vacuole formation were observed.

### 3.2. In Vitro Effects of Cell-Free Filtrate and Antifungal Volatile Compounds of P. variotii on Mycelial Growth of Phytopathogens

**Figure 5:**
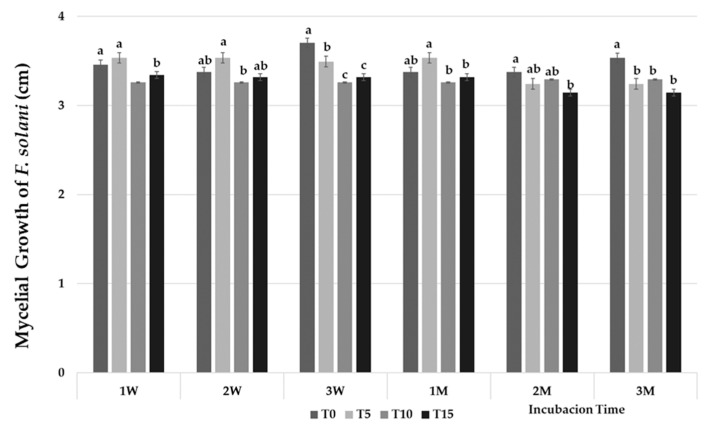
Effect of the cell-free filtrate of *P. variotii* culture on mycelial growth of *F. solani*. Incubation time: 1, 2, and 3 weeks (W); 1, 2, and 3 months (M). T0, T5, T10, T15: % extracts incorporated and mixed with cooled PDA at 0 (Control), 5, 10, and 15% (*v/v*). Means with the same letter are not significantly different (least significant difference test) according to an analysis of variance.

Cell-free filtrate of *P. variotii* did not show any inhibitory effect on mycelial growth of the analysed fungi in the different tested conditions, except for *F. solani*, which showed inhibition ranging from 1.7% to 12% at concentrations of 10% and 15%, regardless of the incubation time (Figure 5).

**Figure 6:**
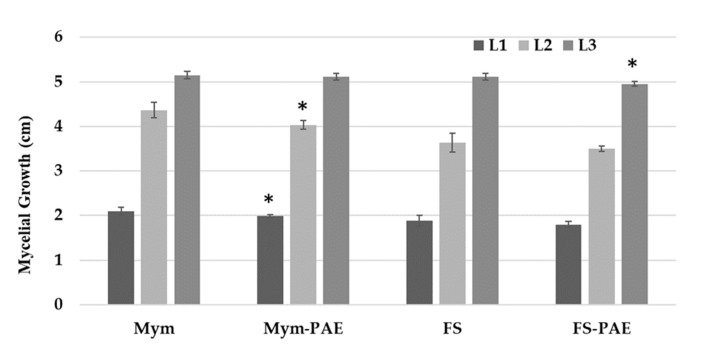
Effect of volatile metabolites of *P. variotii* on mycelial growth of *M. melonis* and *F. solani*. L1, L2, and L3: measurements performed every two days. Data were analysed using Student’s *t*-test. Means with * denote significant difference between both treatments according a pairwise comparison using Student’s t-test (*p* < 0.05).

By contrast, volatile metabolites of *P. variotii* showed no significant inhibition of the tested fungi. In the case of *M. melonis*, low inhibition of 5% and 7% were observed for the L1 and L2 readings (L), respectively. For *F. solani*, 3.2% inhibition was detected in the L3 reading (Figure 6).

### 3.3. Compatibility for Use of P. variotii with Fungicides

The observed effects of 24 fungicides on growth and development of *P. variotii* are presented in Table 1. The results indicate that mycelial growth of *P. variotii* was affected by the different doses of each of the fungicides tested in vitro, compared to the control. According to the OILB scale [57], the compatibility of the 24 fungicides tested for minimum (D2) and maximum (D3) recommended doses in horticultural crops was as follows: 2 were harmless (Pencycuron and Propamocarb; inhibition < 30%), 6 were slightly toxic (diethofencarb, triadimenol, myclobutanil, azoxystrobin, kresoxim-methyl, and Fosetyl-AL; 30–75%), 4 were moderately toxic (thiophanate-methyl, flutriafol, Chlorothalonil, and Cymoxanil; 75–90%), and 10 were toxic (iprodione, tetraconazole, pyrimethanil, etridiazole, copper oxychloride, mancozeb, cyprodinil + fludioxonil, Folpet + metalaxyl-M, dimethomorph + mancozeb, and benalaxil + cymoxanil + mancozeb; >90%). Only two fungicides (fenhexamid and copper hydroxide) showed different behaviour according to the scale at maximum and minimum doses; both were toxic at the maximum doses (D3) and slightly toxic and moderately toxic, respectively, at the minimum doses (D2). The four fungicides formulated with two or three active ingredients were toxic, with 100% mycelial growth inhibition at all tested doses; one mixture (cyprodinil + fludioxonil) was fungistatic, and the other three were fungicidal. Of the remaining six toxic fungicides, total growth inhibition was observed only in three and five at doses D2 and D3, respectively, with fungicidal effects only using pyrimethanil at the minimum dose (D2) and with iprodione, pyrimethanil, and etridiazole at the maximum dose (D3).

### 3.4. Detached Leaves

**Figure 7:**
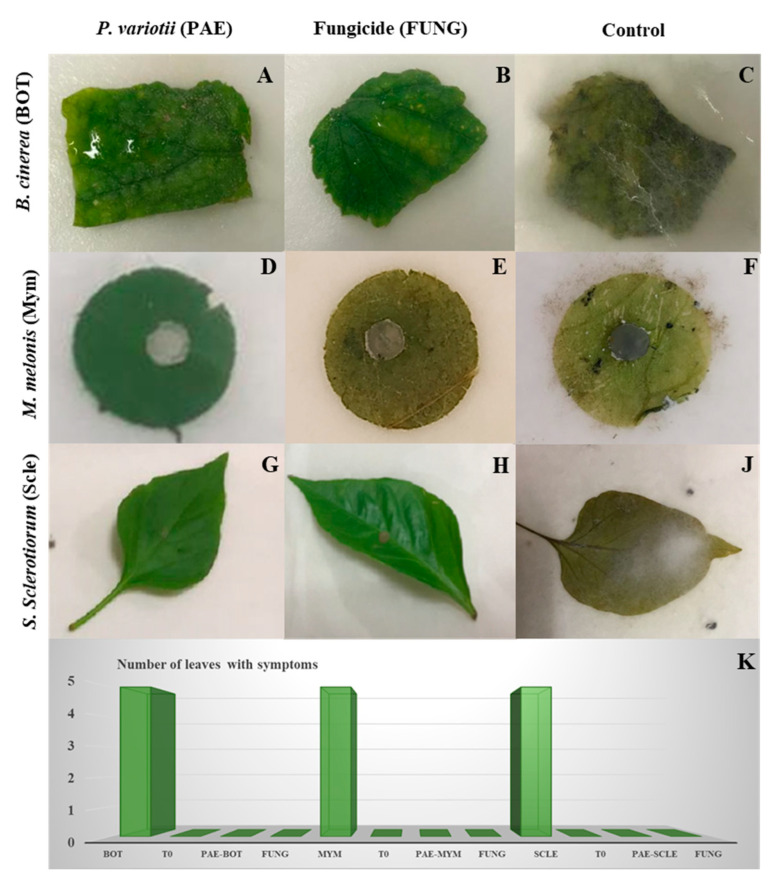
Visual symptoms of *B. cinerea* (**A**–**C**), *M. melonis* (**D**–**F**), and *S. sclerotiorum* (**G**,**H**,**J**) infection on detached leaves treated with *P. variotii* (**A**,**D**,**G**) or fungicide (**B**,**E**,**H**). Control (**C**,**F**,**J**): visual symptoms without *P. variotii* or fungicide. (**K**). Number of leaves with symptoms in each treatment.

Inoculation of detached cucumber, pepper, and tomato leaves with mycelium of *B. cinerea*, *S. sclerotiorum*, and *M. melonis* resulted in marked inhibition of disease development in leaves inoculated with *P. variotii*, at the same efficacy as the control fungicide. Leaves not inoculated with *P. variotii* showed mycelial growth and obvious symptoms of rotting (Figure 7).

### 3.5. In Vivo Suppressive Effects of P. variotii on Diseases Caused by Phytopathogenic Fungi

**Figure 8:**
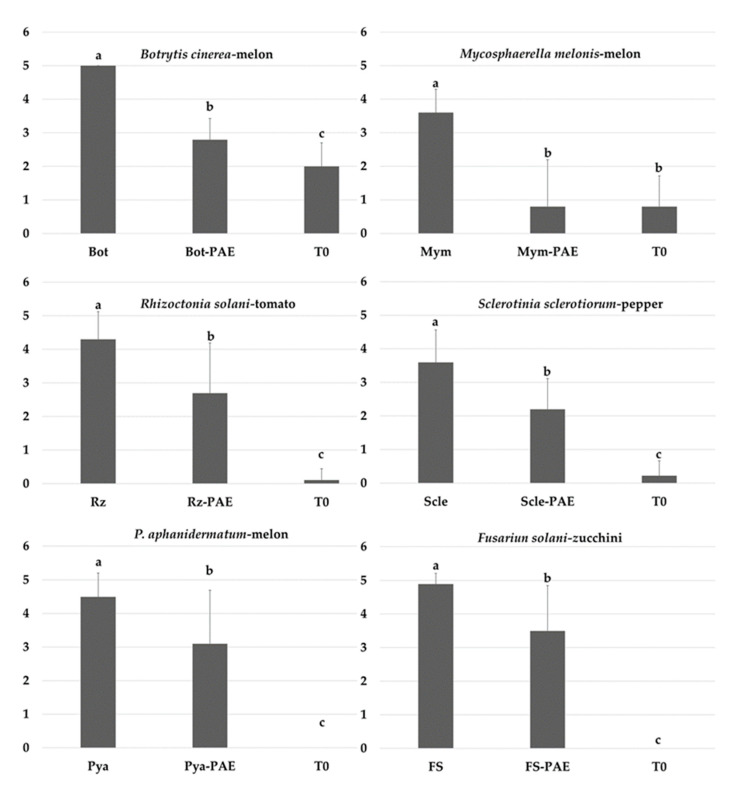
Disease severity of phytopathogens in plants was assessed 30–60 days after inoculation based on a scale from 0 to 5, where 0 indicates no visible disease symptoms and 5 indicates a dead plant. Bars indicate mean standard deviations (24 plants/repetition). Means with the same letter are not significantly different (least significant differences) according to an analysis of variance.

In general, the severity of disease caused by different soil and aerial phytopathogens decreased due to application of *P. variotii* (Figure 8). The strongest biocontrol effect occurred against stem gummosis, with a reduction of 78% in disease severity after infection with by *M. melonis* after spraying with *P. variotii*. Lower values occurred in the other phytopathogen experiments. Reductions of 44%, 38.88%, and 37.20% in disease severity after infection with *B. cinerea*, *S. sclerotiorum*, and *R. solani*, respectively, were observed. Due to the random arrangement of treatments, disease was also observed in the uninoculated controls (T0) of some of the pathosystems. The highest disease severity indices following application of *P. variotii* occurred in the *P. aphanidermatum* and *F. solani* treatments, as opposed to the in vitro results, with a reduction of 31.11% and 28.57%, respectively. In the case of *F. solani*, all plants treated with *P. variotii* reached disease grade 5, showing a delay of approximately 10 days.

**Figure 9:**
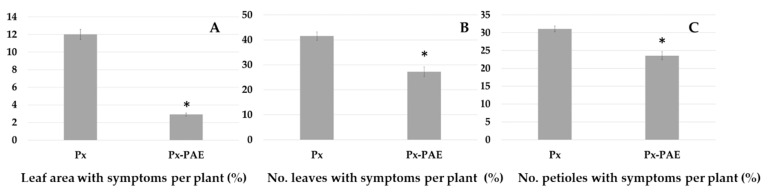
Control of *P. xanthii* by spraying zucchini plants with *P. variotii*. (**A**). Mean proportion of leaf area with powdery mildew symptoms per plant. (**B**). Percentage of the number of leaves with symptoms per plant. (**C**). Percentage of number of petioles with powdery mildew. Px: Application of *P. xanthii*. Px-PAE: Application of *P. variotii* three days before application of *P. xanthii*. Means with * denote significant difference between both treatments according a pairwise comparison using Student’s t-test (*p* < 0.05).

Foliar application of *P. variotii* considerably suppressed the disease caused by *P. xanthii* (Figure 9), with a reduction of 75.80% in the proportion of leaf area showing symptoms. Similarly, the number of leaves and petioles with powdery mildew was reduced by 30.4% and 17.4%, respectively.

## 4. Discussion

*P. variotii* has been described as a biological control agent of fungal and bacterial diseases. Its biocontrol effect is mostly due to its rapid growth and sporulation, resulting in higher competition success for space and available resources compared to other species [60]. However, few studies have conducted in vivo experiments on disease control by *P. variotii* [19].

The current study analysed in vitro and in vivo suppressive effects of *P. variotii* CDG33 on several phytopathogenic fungi that cause diseases of aerial and soil parts of crop plants. In vitro antagonism results based on the dual culture technique showed very heterogeneous values in terms of inhibition percentages, ranging from 25% to 82.0%. Results obtained in in vitro experiments can vary substantially depending on experimental conditions and may differ even between isolates of the same species. Perveen et al. [33] reported similar results from the dual culture of *P. variotii* and *P. aphanidermatum*, with an inhibition percentage of 67–72%, whereas Rodrigo et al. [23] observed lower values that did not exceed 40%. In the present study, inhibition of *P. aphanidermatum* exceeded 80%, which is mainly due to the rapid dispersal of *P. variotii* spores during the experiment. Moreover, substantial inhibition of *F. solani* was observed, which is in line with the results of Ramzan et al. [27]. However, no direct relationship between in vitro and in vivo antagonism values was found, especially regarding *P. aphanidermatum* and *F. solani*. The same was observed in *R. solani* and *M. melonis*, which, despite showing the lowest in vitro antagonism values of no more than 40%, showed the highest disease reduction values in the plant experiments. This has been commonly observed [61,62] and can be caused, among other factors, by differences in techniques and components, such as the culture medium, presence of soil or substrate, and the plant itself. Even so, it is one of the most commonly used techniques and is considered the basis for screening microorganisms as a preliminary step to in vivo experiments, and to produce effective biological control agents in agriculture.

*P. variotii* produces a large number of volatile and non-volatile compounds [19,63,64]; however, little or no inhibition effect of the metabolites produced by isolate CDG33 on the tested phytopathogens was observed. This may be because the effect of volatile and non-volatile metabolites on the tested phytopathogens decreases over time within the first 24–48 h, and the diameter of the fungal colony in the treatments with filtrate was similar to that of the control. Only a mild inhibitory effect of non-volatile metabolites on *F. solani* was observed. Rodrigo et al. [23] obtained similar results, with a significant reduction of *F. moniliforme* with *P. variotii* filtrates. Such a time-dependent decrease in the effect of metabolites on *Biscogniauxia mediterranea*, the causal agent of charcoal disease in cork oak, was described previously [65].

It is important to assess compatibility of biological control agents and agrochemicals by using a combination of chemical and biological tools. Chemical treatments should be based on selection of agrochemicals with the lowest impact on biological control agents. Many studies, even though in clinical settings, confirmed the incompatibility of *P. variotii* with several fungicides, since different species of the genus *Paecilomyces* cause a variety of infections in immunocompromised patients [66,67,68]. Furthermore, previous studies indicate that *Paecilomyces* isolates from agricultural environments are more resistant to common fungicides. This is the case with *P. niveus* isolates from naturally occurring infections in apples, where it can cause post-harvest spoilage and patulin accumulation [69]. Species of the genus *Paecilomyces* may differ regarding their compatibility with fungicides, which may, however, also be the case regarding different strains of the same species due to genetic differences [70,71]. Even though the results obtained in the laboratory may not confirm those of the field experiments due to the higher severity of in vitro assays, our results indicate that *P. variotii* may be compatible with many of the tested fungicides regarding fungistatic effects or reduction of growth rates. Of the 24 tested fungicides, only 4 were incompatible at the minimum recommended doses, and 6 at the maximum doses. Particularly when fungicide mixtures with different modes of action are used, these products may thus interfere with successful biological control by *P. variotii*, which showed 100% fungal inhibition with fungicidal effects on *P. variotii* in three of the tested formulations. Regarding fungicides with one active ingredient, only iprodione, pyrimethanil, and etridiazole acted as fungicides in the authorised dose range. Other products that completely inhibited growth, such as tetraconazole, copper hydroxide, and copper oxychloride, were not biocidal.

Similar results were obtained by Er and Gökçe [72] who applied iprodione at 500 ppm active ingredient (ai) to an isolate of *Paecilomyces fumosoroseus*, even though a reduction of 51% in mycelial growth was observed using a different isolate, Loureiro et al. [73] classified the effect of iprodione on *P. fumosoroseus* as toxic at 500 ppm ai and very toxic at 750 ppm ai; Biango-Daniels et al. [69] analysed the compatibility of 30 isolates of *P. niveus* with the fungicide pyrimethanil to determine the effective fungicide dose at which growth was inhibited by 50% (E_C_50). The E_C_50% values ranged from 29 to 109 and were higher in isolates from crops. In the current study, the lowest tested dose (D1; 100 ppm ai) completely inhibited growth, although without a fungicidal effect which only occurred using higher doses. In contrast, the study of Gallego-Velásquez et al. [74] showed that the application of ethidiazole at 350 ppm ai completely inhibited growth of *P. lilacinum*. In our study, complete inhibition of *P. variotii* growth occurred at dose D3 (1440 ppm ai), and growth inhibition above 92% was observed only at 960 and 480 ppm ai. Other studies showed that copper hydroxide at 1660 and 2592 ppp ai used on *P. fumosoroseus* resulted in growth inhibition of approximately 9% and 82%, respectively [75], while Dermici and Denihan [76] observed a minimum inhibitory concentration of >5000 ppm ai for *P. lilacinus*. Copper oxychloride at 1500 ppm ai completely inhibits growth of *P. lilacinus* [76], however others found inhibition of only 44.1% using copper oxychloride at 4200 ppm ai. In the current study, copper oxychloride completely inhibited growth of *P. variotii* at all doses, and copper hydroxide did the same at the highest dose, thus both compounds were fungistatic. By contrast, Ondráčková et al. [76] observed growth inhibition of 79.6% in *P. lilacinus* treated with mancozeb at 1500 ppm ai; the lowest dose tested in our study was 800 ppm ai, which resulted in >90% inhibition, and it was not fungicidal at any of the tested doses. The fungicides pencycuron and propamocarb were compatible with *P. variotii* at all tested doses. These results corroborate those of previous studies on the effects of pencycuron at 540 ppm ai [77] and propamocarb at 1042.5 ppm ai [74] on *P. lilacinus*.

The present study demonstrated a high capacity of *P. variotii* CDG33 to reduce the severity of diseases caused by different aerial and soil phytopathogens in vivo, even though some pathogens showed very limited inhibition in vitro, according to a previous study [78]. The genus *Paecilomyces* is best known for its role as an entomopathogen and nematophagus fungus; however, other research findings also support the use of *Paecilomyces* fungi to prevent or treat fungal diseases [79]. In this regard, *P. variotii* has been previously described as a BCA in in vivo experiments in which the application of this microbial antagonist reduced colonisation of sunflower seedlings with *M. phaseolina* [28]. Similarly, Maitlo et al. [26] showed a significant reduction in the severity of disease caused by *F. oxysporum* f. sp*. ciceris* from 80% (control) to 6% and 36% following the application of *P. variotii* and *P. lilacinus*, respectively. By contrast, in vitro inhibition percentages of *P. tenuis* were described previously [80], which are similar to those observed in our study, particularly regarding *R. solani* (28%) and *F. oxysporum* (51%), even though lower concentrations of *Pythium* sp. (24%) were used.

No inhibitory effect on phytopathogens through volatile metabolites was observed. A previous study showed in vitro inhibition of *B. cinerea* by approximately 43% due to *P. variotii* [21] which is lower than the inhibition effect exerted by the *P. variotii* isolate CDG33, and this study suggested a significant reduction in rotting of kiwifruit caused by *Botrytis*, which was more effective than the conventional fungicide vinclozolin. Similar in vitro antagonism values (44%) of *Paecilomyces* sp. were observed in a study conducted by Marín-Chacón et al. [81].

## 5. Conclusions

According to our results, the application of the ascomycete *P. variotii* CDG33 as a preventive and/or control of fungal plant diseases may be a viable alternative to the use of conventional synthetic compounds. These insights can help reduce the amount of agrochemicals used which may leak into other ecosystems, and will therefore help reduce the environmental burden of agriculture.

## Figures and Tables

**Table 1:** Effect of different fungicides on mycelial growth of *P. variotii* at different doses (D1-D4). Mean values (± standard deviation) followed by different letters (line) indicate significant differences (*p* < 0.05) by the least significant difference test (LSD). Fungicide action mechanisms (blue numbers): (1) Cytoskeleton and motor proteins, (2) Signal transduction, (3) Sterol biosynthesis in membranas, (4) Amino acids and protein synthesis, (5) Respiration, (6) Lipid synthesis or transport / membrane integrity or function, (7) Chemicals with multi-site activity, (8) Host plant defence induction, (9) Unknown mode of action, (10) Nucleic acids metabolism, and (11) Cell wall biosynthesis.

			Doses (ppm)							
			D1 (0.5 × D2)		D2		D3		D4 (1.5 × D3)	
FUNGICIDE-ACTION MECHANISMS	D2	D3	Growth	Inhib.	Growth	Inhib.	Growth	Inhib.	Growth	Inhib.
Thiophanate-methyl 70% (WP) *w/w*- (1)	500	1000	27.8 ± 2.8 a	60.1%	13.6 ± 1.7 b	80.5%	11.8 ± 1.9 b	83.0%	12.6 ± 1.5 b	81.9%
Diethofencarb 25% (WP) *w/w*- (1)	1000	1500	42.4 ± 2.3 a	41.4%	44.6 ± 1.3 a	38.4%	32.8 ± 2.9 b	54.7%	18.2 ± 1.3 c	74.9%
Pencycuron 25% (SC) *w/v*- (1)	5000	8000	58.2 ± 5.8 a	19.6%	60.8 ± 4.8 a	16.0%	59.2 ± 3.3 a	18.2%	63.2 ± 1.6 a	12.7%
Iprodione 50% (SC) *w/v*- (2)	1000	1500	0 ± 0 a	100.0%	0 ± 0 a	100.0%	0 ± 0 a	100.0%	0 ± 0 a	100.0%
Flutriafol 12.5% (SC) *w/v*- (3)	2000	2500	13.5 ± 0.4 a	78.1%	12.6 ± 2.1 a	79.5%	12.2 ± 1.1 a	80.2%	11.8 ± 1.6 a	80.8%
Triadimenol 25% (EC) *w/v*- (3)	250	500	61 ± 8.6 a	1.0%	36.8 ± 6 b	40.3%	26.8 ± 3 c	56.5%	29 ± 1.9 c	52.9%
Myclobutanil 24% (EC) *w/v*- (3)	200	400	17.6 ± 2.4 a	71.4%	16 ± 1 a	74.0%	15.4 ± 1.1 a,b	75.0%	13.2 ± 1.6 b	78.6%
Tetraconazole 12.5% (ME) *w/v*- (3)	200	400	21 ± 1.6 a	68.7%	4.4 ± 1.3 b	93.4%	0 ± 0 c	100.0%	0 ± 0 c	100.0%
Fenhexamid 50% (WG) *w/w*- (3)	1500	2000	14 ± 2.1 a	77.3%	9.2 ± 0.4 a	85.1%	3 ± 0.7 b	95.1%	3.7 ± 0.7 c	94.0%
Pyrimethanil 40% (SC) P/V- (4)	1500	2000	0 ± 0 a	100.0%	0 ± 0 a	100.0%	0 ± 0 a	100.0%	0 ± 0 a	100.0%
Azoxystrobin 25% (SC) *w/v*- (5)	800	1000	22.2 ± 3.2 a	64.0%	18 ± 2.2 a	70.8%	19.4 ± 1.5 a	68.5%	19.2 ± 2.8 a	68.8%
Kresoxim-methyl 50% (WG) *w/w*- (5)	200	500	31 ± 1.6 a	53.7%	31.4 ± 0.5 a	53.1%	26.8 ± 4.9 b	60.0%	28 ± 2.1 a,b	58.2%
Etridiazole 48% (EC) *w/v*- (6)	2000	3000	5.6 ± 0.9 a	92.3%	1.6 ± 0.5 b	97.8%	0 ± 0 c	100.0%	0 ± 0 c	100.0%
Propamocarb 60.5% (SL) *w/v*- (6)	2500	5000	56.4 ± 4.9 a	22.1%	62.8 ± 6.6 a	13.3%	59.2 ± 3.3 a	18.2%	64.4 ± 2.5 a	11.0%
Copper hydroxide 35% (WG) *w/w*- (7)	2000	3000	29.2 ± 2.2 a	52.6%	20.4 ± 0.9 b	66.9%	0 ± 0 c	100.0%	0 ± 0 c	100.0%
Copper oxychloride 38% (SC) *w/v*- (7)	2000	3000	0 ± 0 a	100.0%	0 ± 0 a	100.0%	0 ± 0 a	100.0%	0 ± 0 a	100.0%
Mancozeb 80% (WG) *w/w*- (7)	2000	3000	6 ± 1.2 a	90.3%	4 ± 0.7 b	93.5%	2.6 ± 0.9 c	95.8%	2.2 ± 0.4 c	96.4%
Chlorothalonil 50% (SC) *w/v*- (7)	2500	3000	13.6 ± 1.8 a	77.9%	13.4 ± 1.8 a	78.2%	15.2 ± 1.8 a	75.3%	15.4 ± 1.5 a	75.0%
Fosetyl-AL 80% (WG) *w/w*- (8)	2500	3000	52.4 ± 1.8 a	14.9%	41.8 ± 1.3 b	32.1%	39.2 ± 2.5 c	36.4%	35.4 ± 1.8 d	42.5%
Cymoxanil 60% (WG) *w/w*- (9)	200	300	36.2 ± 4 a	46.0%	16.2 ± 0.8 b,c	75.8%	17 ± 1.6 b	74.6%	13.2 ± 1.6 c	80.3%
Cyprodinil 37.5% + Fludioxonil 25% (WG) *w/w*- (4 + 2)	600	1000	0 ± 0 a	100.0%	0 ± 0 a	100.0%	0 ± 0 a	100.0%	0 ± 0 a	100.0%
Folpet 40% + Metalaxyl-M 10% (WP) *w/w*- (7 + 10)	2000	2500	0 ± 0 a	100.0%	0 ± 0 a	100.0%	0 ± 0 a	100.0%	0 ± 0 a	100.0%
Dimethomorph 7.5% + Mancozeb 66.7% (WG) *w/w*- (11 + 7)	2000	3000	0 ± 0 a	100.0%	0 ± 0 a	100.0%	0 ± 0 a	100.0%	0 ± 0 a	100.0%
Benalaxil 6% + Cymoxanil 3.2% + Mancozeb 40% (WP) *w/w*- (10 + 9 + 7)	2500	3500	0 ± 0 a	100.0%	0 ± 0 a	100.0%	0 ± 0 a	100.0%	0 ± 0 a	100.0%
